# Prevalence of Non-Alcoholic Fatty Liver Disease in Adult Individuals with Moderate-to-Severe Atopic Dermatitis

**DOI:** 10.3390/jcm12186057

**Published:** 2023-09-19

**Authors:** Martina Maurelli, Paolo Gisondi, Francesco Bellinato, Alessandro Mantovani, Giovanni Targher, Giampiero Girolomoni

**Affiliations:** 1 Section of Dermatology and Venereology, University of Verona, 37129 Verona, Italy; maurelli.martina@gmail.com (M.M.); francesco.bellinato@univr.it (F.B.); giampiero.girolomoni@univr.it (G.G.); 2 Section of Endocrinology, Diabetes and Metabolism, Department of Medicine, University of Verona, 37129 Verona, Italy; alessandro.mantovani@univr.it (A.M.); giovanni.targher@univr.it (G.T.); 3 IRCCS Sacro Cuore Don Calabria Hospital, 37024 Negrar di Valpolicella, Italy

**Keywords:** atopic dermatitis, psoriasis, non-alcoholic fatty liver disease, chronic inflammatory skin diseases

## Abstract

Background: There are no published studies on the prevalence of non-alcoholic fatty liver disease (NAFLD) in patients with atopic dermatitis (AD). Objectives: To estimate the prevalence of NAFLD (assessed via liver ultrasonography) in adults with moderate-to-severe AD. Methods: We performed a retrospective, cross-sectional, observational study including adult patients affected by moderate-to-severe AD, moderate-to-severe chronic plaque psoriasis, or a previous diagnosis of thin melanoma in situ (considered as the control group) who attended the Verona University Hospital between January 2022 and April 2023. Fatty liver was assessed via liver ultrasonography. Results: A total of 144 adults with AD, 466 with chronic plaque psoriasis, and 99 with thin melanoma were included. The prevalence rates of ultrasound-detected NAFLD among patients with in situ melanoma, those with moderate-to-severe AD, and those with moderate-to-severe chronic plaque psoriasis were 23.2% (23 out of 99), 24.1% (36 out of 144), and 49.8% (228 out of 466), respectively (*p* < 0.01). Logistic regression analysis revealed that being of male sex, a higher age, a higher body mass index, and psoriasis were independently associated with NAFLD, whereas AD was not. Conclusions: Our findings show that the prevalence of ultrasound-detected NAFLD in patients with moderate-to-severe AD was comparable to that of patients with a previous diagnosis of in situ melanoma. It is plausible to hypothesize that the Th2-type inflammation typically characterizing AD is not a risk factor for NAFLD. Patients with moderate-to-severe psoriasis, but not those with AD, should be screened for NAFLD and other metabolic comorbidities.

## 1. Introduction

Atopic dermatitis (AD) is a common inflammatory skin disease, affecting nearly 20% of children and adolescents and 2–8% of adult individuals [1]. In most cases, AD develops in early infancy but, in about one-third of cases, AD may also develop in adulthood or in the elderly [1].

AD is a multifactorial skin disease, characterized by defects in the skin barrier allowing the entry of antigens, as well as a tendency to develop Th2-type inflammation [2]. This leads to the abnormal activation of T helper (Th)-2 lymphocytes with a marked overexpression of Th2-type cytokines, such as interleukin (IL)-4, IL-5 IL-13, and IL-31 [3,4]. The clinical presentation of AD is heterogenous. AD typically affects the flexural areas, including the neck, cubital and popliteal fossae, wrists, and ankles. The involvement of the face is also frequent, both in children and adults. Acute AD presents with erythema, edema, scaling, and excoriations. In more severe case, vesicles/papules are also present, with crusting and exudation. Lichenification (i.e., skin thickening) characterizes long-lasting chronic lesions and is more common at folds. Pruritus is the hallmark symptom and constitutes the major part of the disease burden [1]. The itch–scratch cycle contributes to barrier dysfunction and facilitates secondary infections, both cutaneous, such as impetigo and herpes inflection, and extra-cutaneous. The clinical findings vary by patient, ranging from mild to moderate to severe disease. The diagnosis of AD is principally based on clinical findings (including the history, morphology, and distribution of lesions) and associated signs and symptoms [1]. The impact of severe itching and skin lesions on patients frequently leads to a significant impairment in their quality of life [1]. In addition, AD is associated with specific comorbidities, some of which are not restricted to the skin. These comorbidities include other atopic diseases (e.g., asthma and allergic rhinitis), neuropsychiatric disorders, autoimmune diseases, and infections [4]. AD is also commonly associated with elevated levels of immunoglobulin E (IgE). AD is, indeed, associated with several allergic diseases, including food allergies, asthma, and allergic rhinitis [5]. Recently, it has also been reported that patients with moderate-to-severe AD have an increased risk of metabolic syndrome. However, the association of this common inflammatory skin disease with obesity, type 2 diabetes mellitus, hypertension, or dyslipidemia remains uncertain and is still debated [6,7,8,9].

Non-alcoholic fatty liver disease (NAFLD) is the most common cause of chronic liver diseases worldwide, with an estimated global prevalence of around 30% in adults in the general population [10,11,12,13]. NAFLD is characterized by an accumulation of fat in the liver, and is strongly associated with insulin resistance and metabolic syndrome features [14,15,16,17,18]. In particular, NAFLD is considered the hepatic manifestation of the metabolic syndrome and a novel independent risk factor for cardiovascular disease [19,20,21,22]. NAFLD may progress to non-alcoholic steatohepatitis (NASH), cirrhosis and, eventually, hepatocarcinoma [23,24]. Some recent studies have reported that psoriasis is associated with an increased risk of NAFLD, and that this risk parallels the severity of the psoriasis [25]. It is possible to hypothesize that the association between these two disorders is, at least partly, mediated by shared metabolic disorders (such as type 2 diabetes mellitus and obesity), and by a coexisting proinflammatory *milieu*, mainly due to the elevation of IL-1β, IL-6, and tumor necrosis factor-α (TNF-α) [26]. 

As some observational studies have reported that there is a significant association between AD and obesity/metabolic syndrome, and NAFLD is closely associated with metabolic syndrome, it is plausible to assume that an association between AD and NAFLD also exists [14]. Currently, little information is available about the prevalence of NAFLD in patients with AD.

Hence, in this exploratory cross-sectional study, we aimed to examine whether the prevalence of NAFLD (as assessed via ultrasonography) differed in patients with moderate-to-severe AD, compared to patients with moderate-to-severe chronic plaque psoriasis (a patient group at high risk of NAFLD), and those with in situ melanoma (considered as the control group).

## 2. Materials and Methods

We performed a retrospective, cross-sectional study involving adult patients with AD, those with chronic plaque psoriasis, and those with in situ or stage 1 thin melanoma who attended the Dermatology Section of the Verona University Hospital between January 2022 and April 2023. The inclusion criteria of the study were as follows: age >18 years; clinical diagnosis of moderate-to-severe AD, moderate-to-severe chronic plaque psoriasis, or in situ melanoma. Moderate-to-severe AD was diagnosed in the case of an Eczema Area Severity Index (EASI) score >24 [27,28]; moderate-to-severe psoriasis was diagnosed in the case of a Psoriasis Area Severity Index (PASI) score >10, a body surface area (BSA) >10, or a Dermatology Life Quality Index (DLQI) score >10 [29]. The exclusion criteria of the study were as follows: significant alcohol consumption (defined as consumption of more than 20 g of alcohol per day); previous or concomitant treatments with potentially hepatotoxic drugs, such as methotrexate, azathioprine, JAK inhibitors, or cyclosporine; and other coexisting clinical conditions that could induce hepatic steatosis (e.g., viral hepatitis or haemochromatosis). In particular, alcohol abuse, viral hepatitis, and hemochromatosis were considered exclusion criteria for the study, because these conditions are known risk factors for NAFLD.

From all participants, we collected the following clinical and laboratory parameters: age, sex, weight, height, body mass index (BMI), and EASI and PASI scores, as well as a complete blood count, fasting glucose, creatinine, liver enzymes [aspartate aminotransferase (AST), alanine aminotransferase (ALT), and gamma glutamyl transferase (GGT)], and a complete lipid profile. The estimated glomerular filtration rate (eGFR) was calculated using the CKD Epidemiology Collaboration (CKD-EPI) equation. The above-mentioned biochemical blood parameters were measured in all participants after an overnight fast and were assayed, using standard laboratory procedures, at the Central Laboratory of our hospital.

Experienced radiologists, blinded to participants’ clinical and biochemical details, performed liver ultrasonography on all participants. Hepatic steatosis was diagnosed based on characteristic ultrasonographic features, such as a diffuse hyper-echogenicity of the liver relative to the kidneys, ultrasound beam attenuation, and a poor visualization of the intrahepatic vessel borders and diaphragm [30]. Finally, the presence of liver fibrosis was estimated using the BARD score and the fibrosis (FIB)-4 index [31,32]. The FIB-4 index was calculated as follows: age × AST [IU/L]/platelet count [×100,000/L)] × sqrt (ALT [IU/L]). Patients with NAFLD were considered to have indeterminate or advanced fibrosis if their FIB-4 index was ≥1.3 [33]. The BARD score was calculated as BMI ≥28 kg/m^2^ (1 point) + AST/ALT ratio ≥0.8 (2 points) + presence of diabetes (1 point). The two validated risk categories for the BARD score were as follows: high (a score of 2–4—advanced fibrosis likely) or low (a score of 0–1—advanced fibrosis not likely) [33]. 

### 2.1. Statistical Analysis 

The absolute and relative frequency were used to describe the qualitative variables; mean and standard deviation (SD) or median and interquartile range (IQR) were used to describe the quantitative variables. The differences among the three patient groups were tested using the chi-square test for qualitative variables, the one-way ANOVA for quantitative variables with a normal distribution, and the Kruskal–Wallis test for quantitative variables with a skewed distribution, respectively. Direct intergroup comparisons of all variables between patients with melanoma in situ, those with atopic dermatitis, or those with psoriasis were also performed, using adequate post hoc comparison tests. Logistic regression analyses were performed to test the independence of the association between AD and NAFLD (that was included as the dependent variable) after adjusting for age, sex, BMI, and pre-existing diabetes. We have included the aforementioned covariates because they were significant in univariate analyses and represent known risk factors for NAFLD. No interaction terms were formally included in the adjusted regression model. A *p*-value of <0.05 was considered statistically significant. The statistical analyses were performed using STATA software, version 16.0 (STATA, College Station, TX, USA). 

### 2.2. Ethics Approval and Consent to Participate

The present study was conducted in accordance with the Declaration of Helsinki published in 1964 on ethical principles for medical research involving human subjects, and after approval of the study design by the local ethics committee. The ethics committee exempted our research from the need for informed consent from participants because we only retrospectively accessed a de-identified database for data analysis.

## 3. Results

The study included 144 adult patients with moderate-to-severe AD, 466 patients with chronic plaque psoriasis, and 99 patients with in situ melanoma. 

The main clinical and biochemical characteristics of the study participants are reported in Table 1. 

The EASI and PASI values of the study participants were 26.5 ± 5.6 and 15.4 ± 3.6, respectively (data not included in the table). Male sex, age, and BMI were significantly higher in patients with chronic plaque psoriasis than in patients with AD or melanoma. Psoriatic patients were also more likely to have type 2 diabetes mellitus and obesity, and had higher circulating levels of fasting glucose and triglycerides than the other two patient groups. The levels of serum transaminases, eGFR, and total and HDL cholesterol levels were not significantly different among the three groups. In addition, as reported in Table 1, through performing pairwise comparisons (post hoc tests) between each independent group, we observed important differences for the most metabolic variables (such as age, sex, BMI, glucose, and plasma lipid profile) and the prevalence of NAFLD between patients with melanoma in situ and those with psoriasis, as well as between patients with AD and those with psoriasis. 

**Figure 1:**
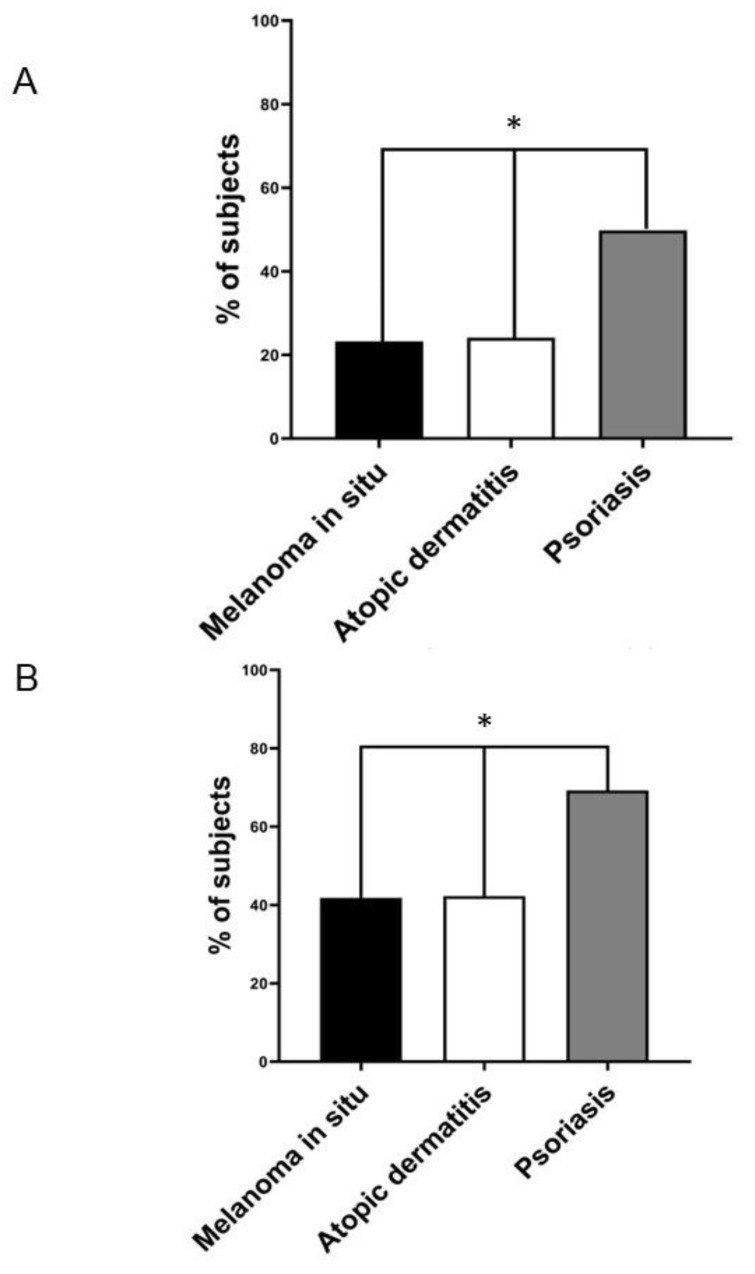
(**A**) The prevalence rates of ultrasound-detected NAFLD in individuals with melanoma in situ (black column, *n* = 99), atopic dermatitis (white column, *n* = 144), and chronic plaque psoriasis (grey column, *n* = 466). (**B**) The prevalence rates of ultrasound-detected NAFLD in the subgroup of patients older than 50 years with melanoma in situ (black column, *n* = 35), atopic dermatitis (white column, *n* = 38), and chronic plaque psoriasis (grey column, *n* = 209). * *p* < 0.01 assessed via the chi-squared test.

Notably, as shown in Figure 1A, the prevalence of NAFLD detected via ultrasonography was remarkably higher in patients with chronic plaque psoriasis than in patients with AD and those with in situ melanoma (49.8% [228 out of 466] vs. 24.1% [36 out of 144] vs. 23.2% [23 out of 99], respectively; *p* < 0.01). In contrast, the prevalence of NAFLD was almost superimposable between patients with AD and those with in situ melanoma. The results remained unchanged even when we restricted the analysis to individuals older than 50 years (Figure 1B). In this case, the prevalence of NAFLD detected via ultrasound was 69.4% [209 out of 301] vs. 42.8% [15 out of 35] vs. 42.1% [16 out of 38] in patients with psoriasis, in situ melanoma, or AD, respectively (*p* < 0.01). Regarding the non-invasive markers of liver fibrosis (as reported in Table 1), the group of patients with psoriasis had a significantly higher AST-to-ALT ratio and a greater proportion of subjects with a FIB4 index equal to or greater than 1.3, or a BARD score ≥2 (indicative of advanced fibrosis). In contrast, these non-invasive markers of advanced liver fibrosis were comparable between patients with AD and those with in situ melanoma.

Table 2 shows the association of AD and the other two skin diseases with ultrasonographically detected NAFLD after adjustment for potential confounders. Logistic regression analysis revealed that being male, a higher age, a higher body mass index, and the presence of chronic plaque psoriasis were independently associated with NAFLD, whereas AD was not (adjusted odds ratio 1.02; 95% CI 0.78–1.26). 

## 4. Discussion

To our knowledge, this is the first exploratory cross-sectional study evaluating the association between moderate-to-severe AD and NAFLD. The novel finding of our cross-sectional study was that the prevalence of NAFLD detected via ultrasonography in adult patients with moderate-to-severe AD was 24.1%, similar to that observed in patients with melanoma in situ (considered as the control group), and to that already reported in the general adult population [10,11,12,13]. The main reasons for which we chose patients with melanoma in situ as the control group are the following: (a) they represent a patient group with a non-inflammatory skin disease, different to psoriasis and AD; (b) based on the current knowledge, it is unlikely that melanoma in situ can significantly influence the development and progression of NAFLD. In addition, and most interestingly, we found a prevalence of ultrasound-detected NAFLD in patients with melanoma in situ that was almost superimposable onto that expected in adults in the general population (about 25–30%) (10–13%).

As expected, we also found that the prevalence of ultrasound-detected NAFLD in patients with moderate-to-severe chronic plaque psoriasis was around 50%, consistent with that already reported in other published studies [34]. We also found that patients with chronic plaque psoriasis were more likely to have advanced liver fibrosis (as estimated from the BARD and FIB-4 scores) than those with AD or thin melanoma. The results of our adjusted regression analysis also confirmed the close association between chronic plaque psoriasis and the risk of prevalent NAFLD. In contrast, AD was not significantly associated with NAFLD after adjusting for age, sex, BMI, and pre-existing type 2 diabetes mellitus.

These data seem to support the notion that the different inflammatory pathways implicated in the pathophysiology of AD and chronic plaque psoriasis might differently affect the risk of having NAFLD. Speculatively, moderate-to-severe AD does not appear to be associated with NAFLD, possibly because the Th2-type inflammation typically characterizing AD does not favor intra-abdominal visceral fat accumulation and ectopic fat deposition in other sites, such as the liver. In contrast, NAFLD is associated with metabolic disorders and chronic plaque psoriasis, which are conditions typically characterized by Th1-type and Th17-type inflammation. Accordingly, a recent meta-analysis examining the association between plasma inflammatory cytokines and NAFLD showed a significant positive association of NAFLD with plasma levels of C-reactive protein, IL-1β, IL-6, TNF-α, and intercellular adhesion molecule-1, while excluding any association with IL-4 [35]. Collectively, our findings further support the notion that patients with chronic plaque psoriasis, particularly those with a moderate-to-severe form, should be screened for NAFLD and other metabolic comorbidities. We cannot relate our findings to other studies because no published studies have assessed the prevalence of NAFLD in adult patients with AD. However, in a recent population-based cohort study, Gau et al. examined the risk of incident AD in patients with NAFLD [36]. These authors reported that the risk of developing AD in patients with NAFLD was significantly lower than that observed in subjects without NAFLD (adjusted hazard ratio 0.93; 95% CI 0.87–0.98). Furthermore, a nearly 20% decrease in the risk of AD was also observed in patients with NAFLD younger than 40 years old [36].

We acknowledge some important limitations of the present exploratory study. In particular, this is a retrospective single-center cross-sectional study that does not allow us to establish any cause-and-effect relationship; the number of patients with AD was relatively small; the diagnosis of NAFLD was based on liver ultrasonography but was not confirmed via liver biopsy or magnetic resonance proton density fat fraction. However, liver ultrasonography has a good specificity and sensitivity for detecting mild-to-moderate hepatic steatosis [37]. Ultrasonography is, therefore, considered the first-line imaging method to diagnose hepatic steatosis in clinical practice. Another limitation of our study is that the generalizability of these findings may be uncertain because it lacks a “pure” healthy adult population as a control group; indeed, as discussed above, in this study, we considered patients with melanoma in situ as a reliable control group. However, in the absence of similar studies, we believe that the data on the prevalence of NAFLD in patients with AD could be valuable because they represent initial evidence, although this needs to be confirmed through further research.

Despite these limitations, our study also has some important strengths, including the completeness of the database, and all patients having been examined (including the liver ultrasound examination), and having had blood samples taken, at the same center.

In conclusion, the results of our exploratory cross-sectional study do not support a significant association between moderate-to-severe AD and NAFLD (assessed via ultrasonography). However, further larger studies are needed to corroborate these findings.

## Figures and Tables

**Table 1:** The main clinical and biochemical characteristics of the study population.

	Melanoma in situ (Group A) (N = 99)	Atopic Dermatitis (Group B) (N = 144)	Psoriasis (Group C) (N = 466)	*p*-Value for Trend ^	*p*-Value for A vs. B *	*p*-Value for A vs. C *	*p*-Value for B vs. C *
Male, *n* (%)	47 (47.47)	72 (50.00)	278 (59.69)	<0.05	n.s.	<0.01	<0.01
Age (years)	53.78 ± 10.69	42.4 ± 11.36	54.05 ± 12.18	<0.05	<0.01	n.s.	<0.01
BMI (kg/m^2^)	24.15 ± 5.92	24.34 ± 6.43	27.06 ± 5.13	<0.01	n.s.	<0.01	<0.01
Obesity, *n* (%)	10 (10.10)	13 (9.03)	121 (25.97)	<0.01	n.s.	<0.01	<0.01
Glucose (mg/dL)	89.23 ± 21.93	90.38 ± 22.39	101.95 ± 44.97	<0.05	n.s.	<0.05	<0.05
Creatinine (mg/dL)	0.79 ± 0.14	0.84 ± 0.22	0.91 ± 0.30	<0.05	n.s.	<0.01	<0.01
eGFR (mL/min/1.73 m^2^)	95.37 ± 21.33	94.32 ± 19.12	91.53 ± 22.12	n.s.	n.s.	n.s.	n.s.
AST (U/L)	22.37 ± 8.27	24.37 ± 9.97	25.35 ± 11.17	n.s.	n.s.	n.s.	n.s.
ALT (U/L)	23.25 ± 23.12	25.15 ± 19.36	28.46 ± 15.8	n.s.	n.s.	n.s.	n.s.
AST/ALT ratio	1.09 ± 0.39	1.14 ± 0.42	1.42 ± 0.23	<0.05	n.s.	<0.01	<0.01
GGT (U/L)	26.03 ± 27.09	27.04 ± 30.19	32.03 ± 29.3	<0.05	n.s.	<0.01	<0.01
Triglycerides (mg/dL)	79.14 ± 69.07	86.12 ± 58	127.4 ± 68.35	<0.05	n.s.	<0.01	<0.01
Total cholesterol (mg/dL)	190.90 ± 49.03	191.19 ± 37.11	192.3 ± 44.15	n.s.	n.s.	n.s.	n.s.
HDL cholesterol (mg/dL)	57.23 ± 13.18	58.15 ± 15.8	53.74 ± 19.56	n.s.	n.s.	n.s.	n.s.
Platelet count (×109/L)	273.47 ± 62.05	270.33 ± 77.95	239.63 ± 59.56	<0.01	n.s.	<0.01	<0.01
Diabetes, *n* (%)	2 (2.02)	2 (1.38)	39 (8.36)	<0.01	n.s.	<0.01	<0.01
NAFLD on ultrasound, *n* (%)	23 (23.23)	36 (24.14)	228 (49.79)	<0.01	n.s.	<0.01	<0.01
BARD score ≥ 2, *n* (%)	26 (26.26)	39 (27.08)	314 (67.38)	<0.01	n.s.	<0.01	<0.01
FIB-4 index ≥ 1.3, *n* (%)	23 (23.23)	29 (20.14)	159 (34.12)	<0.01	n.s.	<0.01	<0.01

Sample size, *n* = 709. Data are expressed as means ± SD or proportions. Abbreviations: BMI, body mass index; NAFLD, non-alcoholic fatty liver disease; AST, aspartate aminotransferase; ALT, alanine aminotransferase; GGT, gamma glutamyl-transferase; FIB-4, fibrosis 4; n.s., not significant. ^ The differences among the three patient groups were tested using the chi-square test for qualitative variables, the one-way ANOVA for quantitative variables with a normal distribution, and the Kruskal–Wallis test for quantitative variables with a skewed distribution, respectively. * Direct inter-group comparisons of all variables between patients with melanoma in situ (Group A), those with atopic dermatitis (Group B), or those with psoriasis (Group C) were performed, using adequate post hoc comparison tests.

**Table 2:** Independent predictors of NAFLD.

	Odds Ratio	95% C.I.	*p*-Value
Age (years)	1.05	1.02–1.06	0.001
Male (yes vs. no)	1.65	1.03–2.64	0.036
BMI (kg/m^2^)	1.25	1.18–1.32	0.001
Type 2 diabetes (yes vs. no)	1.13	0.65–1.94	0.655
Dermatologic diseases			
Melanoma in situ	Ref.	Ref.	
Psoriasis (yes vs. no)	2.86	1.45–5.61	0.002
Atopic dermatitis (yes vs. no)	1.02	0.78–1.26	0.121

Sample size, *n* = 709. Data are expressed as odds ratios and 95% confidence intervals (CI) calculated via logistic regression analysis. The dependent variable of this regression model was the presence of NAFLD detected via ultrasonography. Ref., reference category.

## Data Availability

The data presented in this study are available on request from the corresponding author.
